# Expression profiles of iron transport molecules along the duodenum

**DOI:** 10.1111/jcmm.17313

**Published:** 2022-04-21

**Authors:** Kamila Balusikova, Marketa Dostalikova‐Cimburova, Ilja Tacheci, Jan Kovar

**Affiliations:** ^1^ Department of Biochemistry, Cell and Molecular Biology & Center for Research of Diabetes, Metabolism and Nutrition Third Faculty of Medicine Charles University Prague Czech Republic; ^2^ 2nd Department of Internal Medicine ‐ Gastroenterology University Hospital and Charles University in Hradec Kralove Hradec Kralove Czech Republic

**Keywords:** heme iron absorption, iron transport, iron uptake in duodenum, non‐heme iron absorption

## Abstract

Duodenal biopsies are considered a suitable source of enterocytes for studies of dietary iron absorption. However, the expression level of molecules involved in iron absorption may vary along the length of duodenum. We aimed to determine whether the expression of molecules involved in the absorption of heme and non‐heme iron differs depending on the location in the duodenum. Analysis was performed with samples of duodenal biopsies from 10 individuals with normal iron metabolism. Samples were collected at the following locations: (a) immediately post‐bulbar, (b) 1–2 cm below the papilla of Vater and (c) in the distal duodenum. The gene expression was analyzed at the mRNA and protein level using real‐time PCR and Western blot analysis. At the mRNA level, significantly different expression of HCP1, DMT1, ferroportin and Zip8 was found at individual positions of duodenum. Position‐dependent expression of other molecules, especially of FLVCR1, HMOX1 and HMOX2 was also detected but with no statistical significances. At the protein level, we observed statistically significantly decreasing expression of transporters HCP1, FLVCR1, DMT1, ferroportin, Zip14 and Zip8 with advancing positions of duodenum. Our results are consistent with a gradient of diminishing iron absorption along the duodenum for both heme and non‐heme iron.

## 1 INTRODUCTION

Iron is an essential element for human organism. The total body iron content is up to 5 g of iron per individual (3.5–4 g on average) and only approximately 1–2 mg of that iron is absorbed and simultaneously lost from our organism every day.^1^, ^2^, ^3^ Since body has no mechanism for active iron excretion, the maintenance of iron homeostasis depends on controlled iron absorption.^4^, ^5^

Both heme and non‐heme iron absorption is carried out by enterocytes in the small intestine.^6^, ^7^, ^8^, ^9^ Although heme iron represents only about 20% of total iron in the diet, the efficiency of iron utilization is higher for heme (25–50%) than for non‐heme (1–10%) iron.^10^, ^11^, ^12^, ^13^

For heme absorption into the body, a potential heme transporter across the intestinal apical membrane, heme carrier protein 1 (HCP1) was discovered in 2005.^14^ However, the role of HCP1 as an apical heme transporter has been considered less significant later because of its dual functions as a heme/folate importer with high affinity for folate.^15^ Thus, the mechanism of heme absorption is not fully elucidated yet. Within the cells, heme is metabolized by heme oxygenase‐1 (HMOX1) and ‐2 (HMOX2). Heme metabolized by heme oxygenase produces free ferrous iron that becomes part of the labile iron pool, is stored in a ferritin, or transported from the enterocyte into the bloodstream,^7^, ^16^, ^17^ whereas HMOX1 expression is reported to be inducible, expression of HMOX2 was described as constitutive.^18^, ^19^ However, some findings showed that HO‐2 mRNA expression can also be significantly altered.^20^ Some heme molecules may remain intact within the enterocyte and are subsequently exported from enterocyte either across the basolateral membrane into the circulation or across the apical membrane back into the lumen. The feline leukaemia virus subgroup C cellular receptor 1 (FLVCR1) and ATP‐binding cassette sub‐family G member 2 protein (ABCG2) may be responsible for the respective export activities.^21^, ^22^, ^23^, ^24^

Absorption of non‐heme iron into the body is understood in greater detail and involves the molecules DMT1 (divalent metal transporter 1), ferroportin, Dcytb (duodenal cytochrome b), hephaestin and ceruloplasmin. DMT1 (NRAMP2, DCT1) is a transmembrane iron importer in a mucosal low‐pH environment with preference for divalent iron^25^, ^26^, ^27^, ^28^ while ferroportin (Ireg1, MTP1) mediates ferrous iron export from cells.^29^, ^30^, ^31^ The role of DMT1 is well established, especially in enterocytes, but other metal transporters, such as ZIP8 and ZIP14 (ZRT/IRT‐like protein 8 and 14), have also been identified for non‐transferrin‐bound iron import into the cells.^32^, ^33^ Ferroportin, however, is the only known transporter responsible for iron efflux from cells. Since dietary iron is generally present in its ferric form, ferrireductase Dcytb is involved in the reduction of ferric iron prior to its uptake into the enterocytes via DMT1.^34^, ^35^, ^36^ Iron is utilized within the cell or, after export by ferroportin, it is oxidized by transmembrane ferroxidase hephaestin or plasma ceruloplasmin^37^, ^38^ and bound by plasma transferrin to be distributed within the organism.

Heme and non‐heme iron are absorbed into the organism via enterocytes in the duodenum and proximal jejunum.^6^, ^7^, ^8^, ^9^ However, efficiency of iron absorption may depend on location along the small intestine, most likely controlled by the expression of molecules involved in the process. Regional analyses of iron absorption along the intestinal tract were reported but relied on animal models only.^8^, ^9^ In this study, we characterized the distribution of molecules involved in iron absorption along the human duodenum.

## 2 MATERIALS AND METHODS

### 2.1 Patients

Patients undergoing a gastrointestinal endoscopy as a part of an evaluation of their dyspeptic symptoms and without known disorder of iron homeostasis were enrolled in the study. A total of 10 individuals (6 male, 4 female), mean age of 51.4 years (range 32–65 years) participated. Their iron parameters were within the normal range (serum iron 7–28 μmol/L, serum ferritin for males 20–320 μg/L and for females 20–290 μg/L, and transferrin saturation 20–45%). Hereditary hemochromatosis (HHC), diagnosed by the presence of a risk genotype of the *HFE* gene (i.e., C282Y homozygosity, C282Y/H63D compound heterozygosity or H63D homozygosity), was also excluded. Informed consent was obtained from all participants. The study was approved by the Ethics Committee of the Third Faculty of Medicine, Charles University, Prague and conducted in accordance with the Helsinki Convention.

### 2.2 Sample collection

At gastrointestinal endoscopy, samples were collected at the following locations: (a) immediately post‐bulbar, (b) 1–2 cm below the papilla of Vater and (c) in the distal duodenum. For analysis, samples were stored in RNAlater solution (Sigma‐Aldrich) at −20°C prior to RNA isolation or stored frozen at −80°C prior to protein isolation.

Blood samples were collected prior to endoscopy and processed either for biochemical or DNA analysis and stored short‐term at −20°C.

### 2.3 DNA isolation and PCR‐RFLP

Genomic DNA was isolated from whole blood samples using a QIAamp DNA Blood Mini Kit (Qiagen), according to the manufacturer's instructions.

C282Y, H63D and S65C mutations of the HFE gene were analysed using the PCR‐RFLP method, as described previously.^39^

### 2.4 RNA isolation and real‐time quantitative polymerase chain reaction

Total RNA was isolated from duodenal biopsies using an RNeasy MiniKit along with DNase Set (Qiagen), according to the manufacturer's instructions. The integrity of the RNA was estimated using gel electrophoresis and sample concentrations were measured using a NanoPhotometer (Implen).

Isolated RNA was reverse transcribed using a TaqMan Reverse Transcription Reagents kit (Applied Biosystems) with random primers, according to the manufacturer's instructions. Analogically, the negative controls were prepared when reverse transcriptase was omitted in the reverse transcription reactions. Real‐time quantitative PCR was performed using TaqMan™ Gene Expression Master Mix, TaqMan™ Fast Advanced Master Mix, and Power Sybr™ Green PCR Master Mix (Applied Biosystems). For amplification of cDNA, Applied Biosystems pre‐designed gene expression assays were used for following of tested genes: *HCP1* (Hs00560565_m1), *FLVCR1* (Hs00171953_m1), *HMOX1* (Hs01110250_m1), *HMOX2* (Hs01558390_m1), *FPN1* (Hs00205888_m1), *Zip8* (Hs01061804_g1), *Zip14* (Hs00299262_m1) and housekeeping gene *GAPDH* (Hs99999905_m1). For amplification of *DMT1‐IRE*,^40^ *Dcytb* and *hephaestin* cDNA, previously described primers were used.^41^ Amplification conditions were as follows: initial denaturation at 50°C for 2 min, then 95°C for 10 min, followed by 40 amplification cycles at 95°C for 15 s and 60°C for 1 min. Each sample was analysed in triplicate. All data were normalized to the amount of *GAPDH* cDNA in the sample with the average C*_t_* values being as follows: *HCP1* – 33.5, *HMOX1* – 31.3, *HMOX2* – 35.2, *DMT1* – 32.0, *Dcytb* – 26.3, *ZIP14* – 32.0, *ZIP8* – 34.8, *FLVCR* – 33.9, *FPN1* – 26.4, *HEPH* – 25.7 and *GAPDH* – 23.9. No sample was under detection limit of the method. To exclude any possible genomic DNA contamination affecting our results, the negative control samples of reverse transcription were analyzed when Power Sybr™ Green PCR Master Mix was involved. As for TaqMan^®^ Assays, all employed probes span exons. The ΔΔCq method was used to calculate relative changes in gene expression.^42^

### 2.5 Western blot analysis

After protein extraction from duodenal biopsies using RIPA buffer (Sigma‐Aldrich) containing inhibitors of proteases and phosphatases (Roche), protein concentrations were measured in all samples (Thermo Scientific Pierce BCA Protein Assay Kit, Thermo Fisher Scientific Inc.). The total yield of protein was about 10 µg/µl on average. Based on thorough optimization, the samples for analysis were diluted under reduced conditions to load 20 µg of protein per well and heated at 95°C for 8 min. All samples were heated even though the general recommendations are to not boil samples for ferroportin and DMT1 detection. This recommendation cannot be followed in the case of duodenal biopsies because of the problems with protein separation due to the presence of residual mucin in unboiled samples. In addition, denaturation at 95°C resulted in better visualization of ferroportin and DMT1 signal in our samples.

Western blot analyses of tested molecules (HCP1, FLVCR1, HMOX1, HMOX2, DMT1, Zip8, Zip14, ferroportin, Dcytb and hephaestin) and actin (loading control) were performed as previously described^43^, ^44^ with minor modifications: Proteins were first separated by SDS‐PAGE using a Criterion Cell (Bio‐Rad, Hercules, CA, USA) and then blotted onto a 0.2 μm nitrocellulose membrane for 3 h at 80V, using the Criterion Wire Blotter System (Bio‐Rad). The membrane was blocked with 5% BSA in TBS (100 mM Tris‐HCl, 150 mM NaCl, pH = 7.5). Washed membranes were incubated with the relevant human primary antibody overnight at 4°C. Primary antibodies were as follows: Anti‐HCP1 (ab25134, Abcam), Anti‐FLVCR (H00028982‐D01P, Abnova), Anti‐HMOX1 (ab68477, Abcam), Anti‐HMOX2 (MAB3170, Sigma‐Aldrich), Anti‐DMT1 (SAB2102164, Sigma‐Aldrich), Anti‐FPN1 (ab85370, Abcam), Anti‐Dcytb (HPA014757, Sigma‐Aldrich), Anti‐Hephaestin (ab56729, Abcam), Anti‐Zip8 (HPA038833, Sigma‐Aldrich), Anti‐Zip14 (ab191199, Abcam) and Anti‐Actin, (A3853, Sigma‐Aldrich).

Subsequently, membranes washed off of primary antibodies were incubated for 2 h at room temperature with the corresponding horseradish peroxidase‐conjugated secondary antibody. Goat anti‐mouse IgG‐HRP (sc‐2005) and goat anti‐rabbit IgG‐HRP (sc‐2004) from Santa Cruz Biotechnology (Dallas, TX, USA) were used. Signal was detected by enhanced chemiluminescence using SuperSignal™ West Pico PLUS Chemiluminescent Substrate (Pierce) and a Gel Logic 4000 PRO Imaging System (Carestream Health). Band intensities were quantified by densitometry, and the data were analysed using Carestream Molecular Imaging Software v5.2 (Carestream Health). Data were normalized to actin and related to internal standards (samples from three control patients were present on every immunoblot).

### 2.6 Statistical analysis

Data are presented as means ± SEM. The comparisons of data for individual levels of duodenum (a vs b, a vs c, B vs C – see ‘Sample collection’) were determined using the paired *t*‐test. All tests were two‐tailed and *p* values less than 0.05 (*) and 0.01 (**) were considered significant at 5% and 1% respectively. Statistical analyses were performed using GraphPad Prism (GraphPad Software, Inc., Version 6.00).

## 3 RESULTS

### 3.1 Characteristics of patients

Hematological parameters documenting the iron metabolism status of tested individuals are shown in Table 1. The presence of a risk genotype of the *HFE* gene (diagnosis of hereditary hemochromatosis) was also assessed. No patient was found to have any of the risk genotypes (i.e., C282Y homozygosity, C282Y/H63D compound heterozygosity or H63D homozygosity).

**TABLE 1:** Hematological and baseline characteristics of tested individuals

Parameter	All *N* = 10	Men *N* = 6	Women *N* = 4
Age [yrs]	51.4 ± 3.69	51.0 ± 4.45	52.0 ± 7.22
BMI [kg/m^2^]	24.4 ± 1.12	25.3 ± 1.71	23.0 ± 1.03
Serum Fe [μmol/l]	20.1 ± 1.85	21.4 ± 2.58	18.3 ± 2.66
Serum Ft [μg/l]	177.0 ± 49.48	263.0 ± 59.45	48.1 ± 17.01
Tf saturation [%]	36.9 ± 3.32	40.4 ± 4.73	31.6 ± 3.34
Hb [g/dl]	14.4 ± 0.36	15.1 ± 0.27	13.3 ± 0.35
Ht [%]	42.4 ± 1.02	44.5 ± 0.74	39.3 ± 1.08

Note

Data are presented as arithmetic mean ± SEM. Normal ranges: serum Fe (11–26 μmol/L), serum Ft (male 30–320 μg/L, female 30–290 μg/L), Tf saturation (20–45%), Hb (male 13.0–18.0 g/dl, female 11.5–16.0 g/dl), Ht (male 38–54%, female 35–47%).

Abbreviations: BMI, body mass index; Ft, ferritin; Hb, haemoglobin; Ht, hematocrit; Tf, transferrin.

### 3.2 Expression analysis

**FIGURE 1:**
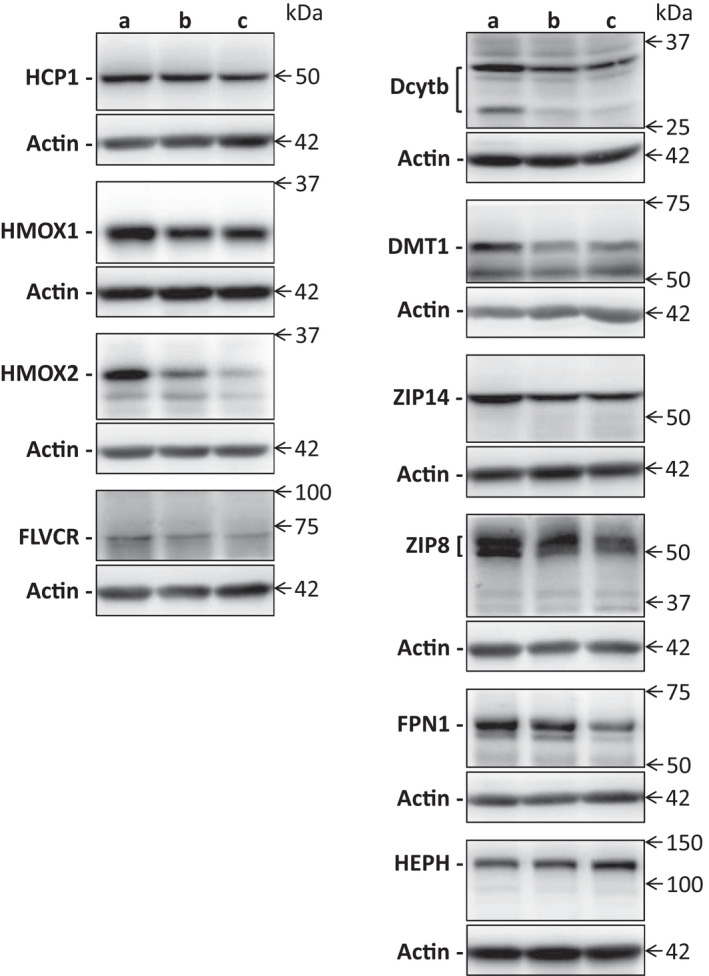
Western blot analysis of HCP1, HMOX1, HMOX2, FLVCR, Dcytb, DMT1, ZIP14, ZIP8, FPN1, HEPH and Actin (loading control). Representative immunoblots comparing expression of tested molecules in duodenal samples of healthy individuals at different levels of duodenum: (a) immediately post‐bulbar, (b) 1–2 cm below the Vater papilla and (c) at the distal duodenum; are shown

The mRNA and protein levels of HCP1, HMOX1, HMOX2, DMT1, Dcytb, Zip14, Zip8, FLVCR1, ferroportin and hephaestin were determined in duodenal samples of tested subjects using quantitative real‐time PCR and Western blot analysis. Representative immunoblots of all tested molecules are shown in Figure 1.

#### 3.2.1 Molecules of iron import into enterocyte

**FIGURE 2:**
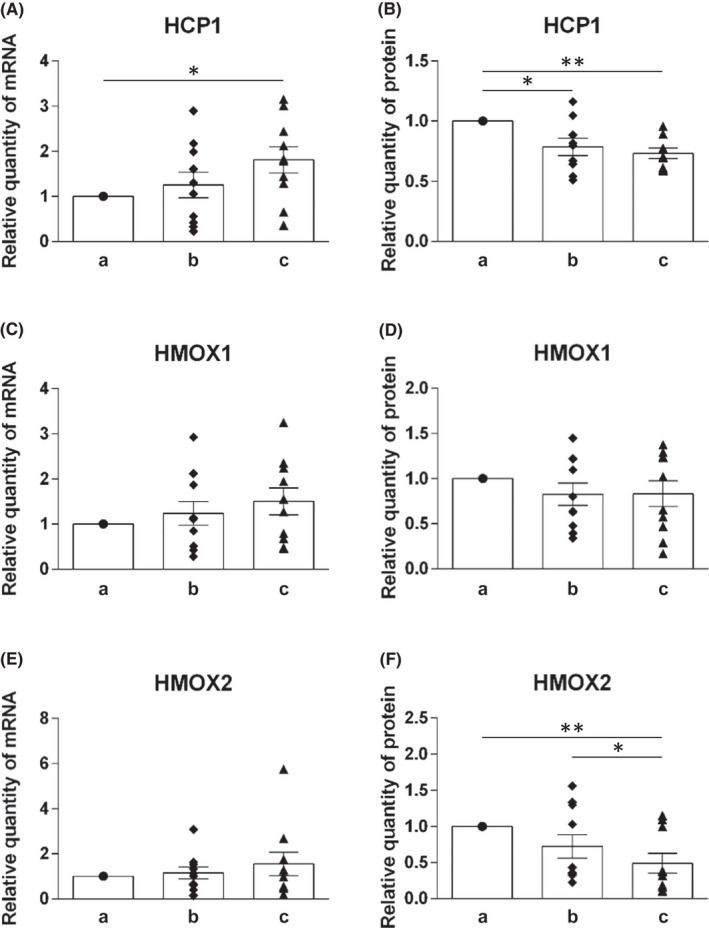
Expression profile of HCP1, HMOX1 and HMOX2 on mRNA and protein levels in duodenal samples of healthy individuals at different levels of duodenum: (a) immediately post‐bulbar, (b) 1–2 cm below the Vater papilla and (c) at the distal duodenum. mRNA levels (A, C, E) were determined using quantitative real‐time PCR, protein levels (B, D, F) were determined using Western blot analysis. Values are presented as relative numbers when comparing with the expression in immediately post‐bulbar duodenum, that is, the column a (1.0). Mean values ± SEM are depicted for b and c. Statistically significant differences (*t*‐test) are indicated by *(*p* < 0.05), **(*p* < 0.01)

**FIGURE 3:**
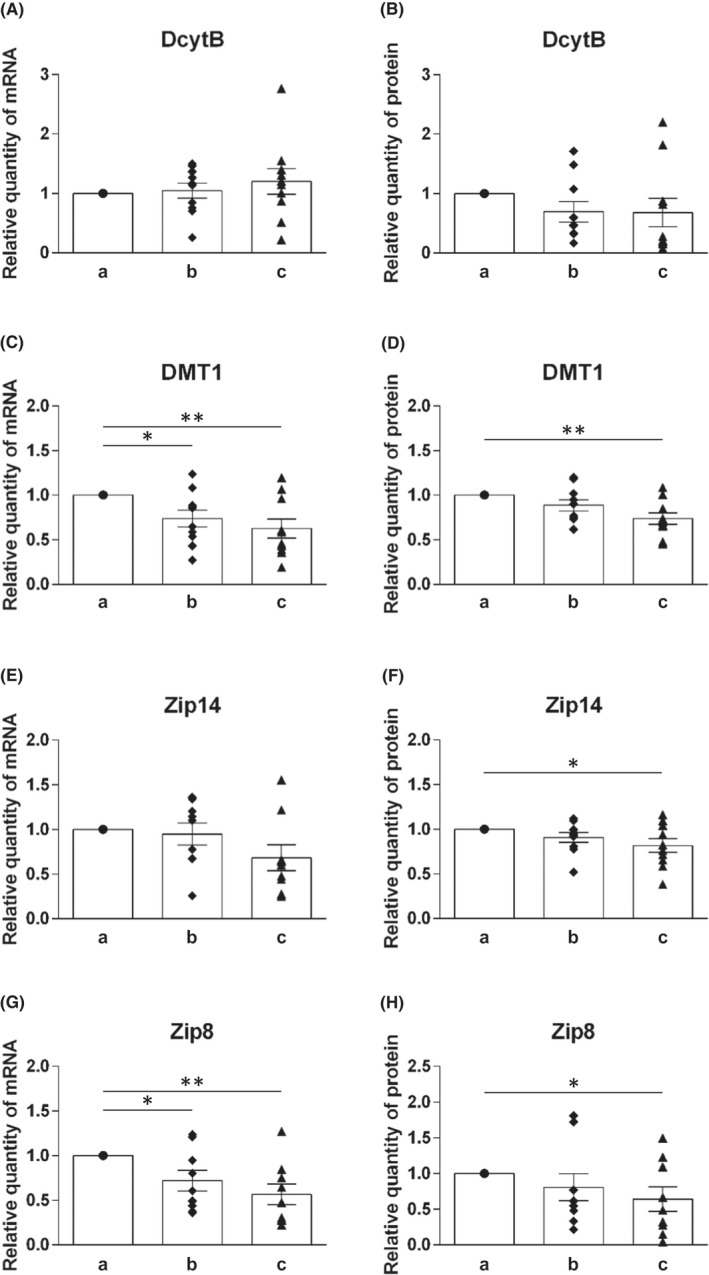
Expression profile of Dcytb, DMT1, ZIP14 and ZIP8 on mRNA and protein levels in duodenal samples of healthy individuals at different levels of duodenum: (a) immediately post‐bulbar, (b) 1–2 cm below the Vater papilla and (**c**) at the distal duodenum. mRNA levels (A, C, E, G) were determined using quantitative real‐time PCR, protein levels (B, D, F, H) were determined using Western blot analysis. Values are presented as relative numbers when comparing with the expression in immediately post‐bulbar duodenum, that is, the column a (1.0). Mean values ± SEM are depicted for b and c. Statistically significant differences (*t*‐test) are indicated by *(*p* < 0.05), **(*p* < 0.01)

The mRNA and protein levels of molecules involved in the uptake of heme iron (HCP1, HMOX1 and HMOX2) and non‐heme iron (DMT1, Dcytb, Zip14 and Zip8) were analyzed (Figures 2 and 3).

##### Molecules of heme iron uptake

Concerning mRNA level, we detected gradually increased expression for all tested molecules with successive position of duodenum. However, this increase was found to be statistically significant only for HCP1 mRNA level when compared (a) and (c) position (Figure 2A).

However, the expression of the corresponding proteins was found to be decreasing along the length of duodenum.

For HCP1 and HMOX2, we detected significantly decreased protein expression (*p* < 0.01) when comparing position (a) and (c) (Figure 2B,F). Moreover, significant decrease in protein expression (*p* < 0.05) for HCP1 was shown between position (a) and (b) (Figure 2B) and for HMOX2 between position (b) and (C) (Figure 2F). Protein expression of HMOX1 at different positions of duodenum seems to be following the trend but without statistical significance (Figure 2D).

Overall, all tested molecules involved in absorption of heme iron into enterocyte seem to have a parallel expression pattern along the length of duodenum. However, there was a discrepancy between mRNA (increase of the expression) and protein level (decrease of the expression).

##### Molecules of non‐heme iron uptake

Expression of Dcytb, involved in redox reactions in order to enable iron availability for transport, was only slightly changed on both mRNA as well as protein levels (Figure 3A,B). Although statistically non‐significant, changes in its expression on mRNA and protein levels were in opposite direction.

Regarding non‐heme iron transporters, that is, primarily DMT1 and also Zip14 and Zip8, all tested molecules had the same expression pattern at both mRNA and protein levels, decreasing along the duodenum.

Expression of DMT1 is significantly decreased (*p* < 0.01) when compared (a) and (c) position in duodenum on mRNA as well as protein level (Figure 3C,D). In addition, we detected significant decrease in expression of DMT1 mRNA (*p* < 0.05) between position (a) and (b) (Figure 3C).

Analysis of Zip 14 and Zip8 expression showed the biggest change for Zip 8 mRNA level. Decrease (*p* < 0.01) between (a) and (c) position in duodenum was accompanied with slightly smaller but still significant decrease (*p* < 0.05) between positions (a) and (b) (Figure 3G). mRNA level of Zip14 was also slightly decreased but without statistical significance (Figure 3E). However, for both molecules, some statistically significant changes (*p* < 0.05) were detected on the protein level when compared expression at (a) and (c) position of duodenum (Figure 3F,H).

#### 3.2.2 Molecules of iron export from enterocyte

**FIGURE 4:**
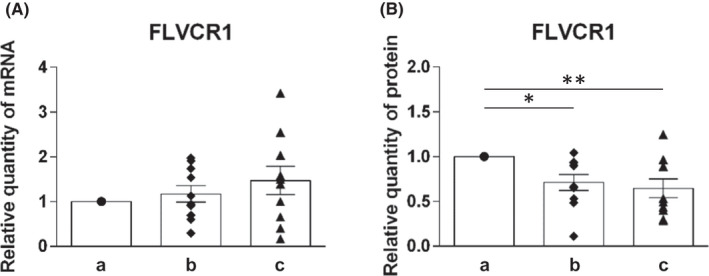
Expression profile of FLVCR1 on mRNA and protein levels in duodenal samples of healthy individuals at different levels of duodenum: (a) immediately post‐bulbar, (b) 1–2 cm below the Vater papilla and (c) at the distal duodenum. mRNA levels (A) were determined using quantitative real‐time PCR, protein levels (B) were determined using Western blot analysis. Values are presented as relative numbers when comparing with the expression in immediately post‐bulbar duodenum, that is, the column a (1.0). Mean values ± SEM are depicted for b and c. Statistically significant differences (*t*‐test) are indicated by *(*p* < 0.05), **(*p* < 0.01)

**FIGURE 5:**
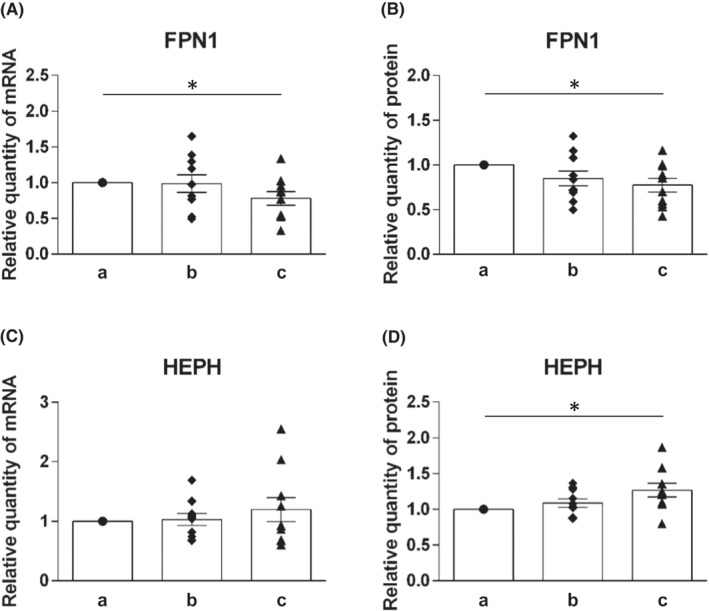
Expression profile of FPN1 and HEPH on mRNA and protein levels in duodenal samples of healthy individuals at different levels of duodenum: (a) immediately post‐bulbar, (b) 1–2 cm below the Vater papilla and (c) at the distal duodenum. mRNA levels (A, C) were determined using quantitative real‐time PCR, protein levels (B, D) were determined using Western blot analysis. Values are presented as relative numbers when comparing with the expression in immediately post‐bulbar duodenum, that is, the column a (1.0). Mean values ±SEM are depicted for b and c. Statistically significant differences (*t*‐test) are indicated by *(*p* < 0.05)

The mRNA and protein levels of molecules involved in efflux of heme iron (FLVCR1) and non‐heme iron (ferroportin and hephaestin) were analysed (Figures 4 and 5).

##### Molecules of heme iron efflux

Concerning mRNA level, we detected gradually increased expression for FLVCR1 with successive position of duodenum. However, this increase was not statistically significant (Figure 4A).

On the contrary, FLVCR1 protein expression was shown to be decreasing along the length of duodenum. We detected statistically significant decrease of FLVCR1 protein expression when comparing position (a) and (b) (*p* < 0.05) and even more pronounced between positions (a) and (c) (*p* < 0.01) (Figure 4B). The expression pattern of FLVCR1 paralleled that of molecules of heme absorption (Figure 2).

##### Molecules of non‐heme iron efflux

The mRNA as well as protein level of ferroportin showed similar expression pattern as that of iron importers. All transporters decreased along the duodenum. For ferroportin, the decrease in its expression was less pronounced but also significant (*p* < 0.05) at both mRNA and protein levels when comparing (a) and (c) position in duodenum (Figure 5A,B).

Expression of hephaestin, molecule involved in in iron oxidation prior to iron distribution to the body, was only slightly increased on both mRNA as well as protein levels. Statistical significance (*p* < 0.05) was found for the difference in hephaestin protein expression between position (a) and (c) (Figure 5D).

#### 3.2.3 Ratio of protein/mRNA expression profile

**FIGURE 6:**
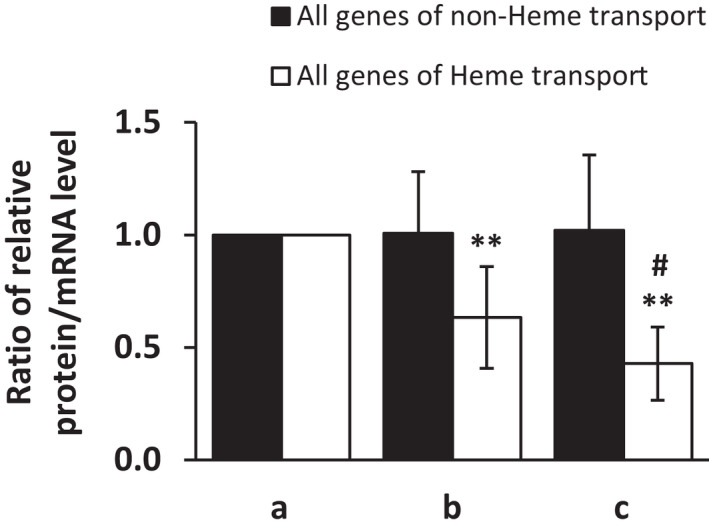
Protein/mRNA ratio of expression profile of non‐Heme transport molecules and Heme transport molecules in duodenal samples of healthy individuals at different levels of duodenum: (a) immediately post‐bulbar, (b) 1–2 cm below the Vater papilla and (c) at the distal duodenum. Values are presented as relative numbers of all genes combined when comparing with the expression in immediately post‐bulbar duodenum, that is, the column a (1.0). Results are depicted as means ± SEM for b and c. Statistically significant differences (*t*‐test) are indicated by **(*p* < 0.01) when compared with a; and by **#**(*p* < 0.05) when compared c vs b

We also analyzed the ratio of protein vs. mRNA expression in individual positions of duodenum (Figure 6). The expression ratio for molecules involved in heme iron transport (import and export as well) is significantly (*p* < 0.01) decreased along the duodenum when position (b) and (c) is compared with (a). Moreover, slightly less significant (*p* < 0.05) decrease was detected between position (b) and (C). However, the ratio is not changed for non‐heme iron transport molecules (importers and exporters as well) when compared different positions in duodenum.

## 4 DISCUSSION

It was shown in animal models that iron absorption can differ along the intestinal tract,^8^, ^9^ a common pattern for transporters of nutrients and drugs in animals and humans.^45^, ^46^, ^47^, ^48^ In this human study, we aimed to assess the expression pattern of molecules involved in the absorption of heme and non‐heme iron into the body along the length of duodenum.

Heme iron absorption as a major source of bioavailable iron for the human body was studied extensively.^6^, ^49^, ^50^, ^51^ However, the main mechanism of heme import into enterocytes is not yet known. The involvement of the transporter HCP1 and heme oxidases HMOX1 and HMOX2 (heme oxygenase 1 and 2) and heme exporter FLVCR1 has been described.^7^, ^14^, ^15^, ^16^, ^17^, ^21^, ^22^, ^23^ As for non‐heme iron absorption, transporters DMT1 and ferroportin with recent addition of ZIP14 and ZIP8 are extensively studied together with iron reductase Dcytb and iron oxidase hephaestin.^25^, ^26^, ^27^, ^28^, ^29^, ^30^, ^31^, ^32^, ^34^, ^35^, ^36^, ^37^, ^52^

As mentioned earlier, iron uptake in digestive tract of animal models was shown to be decreased in the distal direction along the duodenum or when compared in duodenum vs proximal jejunum.^8^, ^9^ Our data indicate that there may be a gradient of iron absorption in human duodenum as well.

Despite differing significance, protein expression of all molecules but hephaestin is decreased distally along duodenum. These results support the idea that the optimal site of iron absorption is the proximal duodenum. It is possible that the decreasing expression of tested molecules distally along the digestive tract is the simple regulatory or developmental consequence of decreased iron availability for transport. In our subjects, individuals with ‘healthy’ iron metabolism, expression pattern of molecules of iron import into the enterocyte is accompanied by a similar expression profile of cellular iron exporters. It is not clear whether pathological disturbances in iron transport disturb this relationship.

The relationship between mRNA and protein expression showed positive correlation only for molecules involved in non‐heme iron transport but anticorrelated for those involved in heme transport. These differences might results from different post‐transcriptional regulation of the molecules. While initiation and inhibition of translation of molecules involved in non‐heme iron transport is mostly affected by iron regulatory protein (IRP) and iron‐responsive element (IRE) interactions,^53^, ^54^ in the case of heme iron transport an HIF/HRE interaction are more likely involved.^52^ Moreover, the expression ratio protein/mRNA for heme transport molecules significantly decreases with further positions of duodenum. The regulatory mechanisms and functional adaptations that drive these differences are not known.

Our analysis of expression profiles of molecules involved in heme and non‐heme iron transport in duodenum are in agreement with previously analyzed animal models. Thus, absorption of iron into the organism via duodenal enterocytes is decreased distally along the duodenum and the most important position for iron absorption in healthy individuals is the proximal duodenum. This study, according to our knowledge, is the first of its type involving samples from humans.

## CONFLICT OF INTEREST

The authors confirm that there are no conflicts of interest.

## AUTHOR CONTRIBUTIONS

**Kamila Balusikova:** Data curation (equal); Formal analysis (equal); Investigation (equal); Methodology (equal); Writing – original draft (lead). **Marketa Dostalikova‐Cimburova:** Conceptualization (equal); Data curation (equal); Formal analysis (equal); Investigation (equal); Methodology (equal). **Ilja Tacheci:** Investigation (equal); Methodology (equal). **Jan Kovar:** Conceptualization (equal); Supervision (lead); Writing – review & editing (lead).

## Data Availability

The data are available on request from the authors.
